# An Assessment of Health Behavior Peer Effects in Peking University Dormitories: A Randomized Cluster-Assignment Design for Interference

**DOI:** 10.1371/journal.pone.0075009

**Published:** 2013-09-09

**Authors:** Changzheng Yuan, Jun Lv, Tyler J. VanderWeele

**Affiliations:** 1 Department of Nutrition, Harvard School of Public Health, Boston, Massachusetts, United States of America; 2 Department of Epidemiology, Harvard School of Public Health, Boston, Massachusetts, United States of America; 3 Department of Epidemiology and Biostatistics, Peking University School of Public Health, Beijing, China; 4 Department of Biostatistics, Harvard School of Public Health, Boston, Massachusetts, United States of America; Chancellor College, University of Malawi, Malawi

## Abstract

**Background:**

Relatively little is known about the peer influence in health behaviors within university dormitory rooms. Moreover, in China, the problem of unhealthy behaviors among university students has not yet been sufficiently recognized. We thus investigated health behavior peer influence in Peking University dormitories utilizing a randomized cluster-assignment design.

**Methods:**

Study design: Cross-sectional in-dormitory survey. Study population: Current students from Peking University Health Science Center from April to June, 2009. Measurement: Self-reported questionnaire on health behaviors: physical activity (including bicycling), dietary intake and tobacco use.

**Results:**

Use of bicycle, moderate-intensity exercise, frequency of sweet food and soybean milk intake, frequency of roasted/baked/toasted food intake were behaviors significantly or marginally significantly affected by peer influence.

**Conclusion:**

Health behavior peer effects exist within dormitory rooms among university students. This could provide guidance on room assignment, or inform intervention programs. Examining these may demand attention from university administrators and policy makers.

## Introduction

Non-communicable disease (NCDs), such as obesity, diabetes, hypertension and cardiovascular disease have become a major public health problem in China, accounting for 80% of deaths and 70% of disability adjusted life-years lost, resulting in enormous economic burden. High health care cost and medical expenditure related to NCDs can moreover lead to poverty and health disparities [1]. The prevalence of childhood overweight and obesity in China has already reached 30% in urban areas [2], [3]; type 2 diabetes prevalence has almost reached 10% [4]; and 30% of Chinese adults have hypertension [5]. It was projected that the percentage of people in China aged 65 or older will reach almost 25% in the next 40 years [6], which could make the prevalence of health-related problems worse. There is now well documented evidence that physical inactivity, unhealthy diets, and tobacco use are three of the main modifiable risk factors for NCDs, and those factors have to be dealt with at population level through societal approaches and policy initiatives [7].

Besides the personal behavioral risk factors above, it has been argued that some public health approaches are ‘ignoring the wider environment within which risk factors arise and thus providing a limited and biased view of disease causation from a population perspective’ [8], [9]. Several studies focused on the environment risk factors have showed that neighborhood peers can have profound effects on both adults and children [10]. When the outcome of one person depends on another's state or exposure such phenomena are sometimes referred to as “interference” or “social influence” or “peer effects”. However, studies that evaluate the social influence of friends or family members are problematic due to the non-random selection of such relationships [11].

When associations between the outcomes or states of peers exist, there are several possible explanations. One is that individuals generally self-select into neighborhoods or roommate pairs with the preference to be around others with similar characteristics, which is also called homophily, resulting in selection bias [12], [13], [14]. This bias can be partially controlled by lagged measurements or can be avoided by randomized designs. The second explanation for associations among peers is potential confounding by shared environmental factor [12] (e.g. the distance to gym, playground and food stores). Ways of dealing with this confounding include control for observable environmental variables. A final explanation is that of “social influence” or “peer effects”, which is the focus of the present investigation. Researchers would like to estimate the peer effects independent of the associations arising from homophily and environmental confounding.

Many studies have revealed peer effects in behaviors including smoking and drug use among adolescents [15], [16], [17], [18] and peer effects in HIV-related unsafe behaviors [19], [20]. However, these may be subject to selection bias or confounding mentioned earlier. After utilizing randomized designs to avoid selection bias and to control for potential confounding, earlier studies also investigated peer effect in academic achievement [21], [22] and weight gain [23] among college students. However, there has been little research focused on health behavior peer effects among college/university students.

To the best of our knowledge, no study has directly examined peer effects in health behaviors within dormitory rooms among university students. Moreover, in China, the problem of unhealthy behaviors among university students has not yet been sufficiently recognized. Given this gap in knowledge, it is of importance to evaluate health behavior peer effects among university students. Freshmen entering Peking University Health Science Center are randomly assigned to dormitory rooms based on sex and majors, and our study will utilize this randomization to assess peer effects in a variety of health behaviors among students at Peking University Health Science Center.

## Materials and Methods

### Ethics Statement

The study was conducted between April and June in 2009 in China. The Institutional Review Board of the Harvard School of Public Health has affirmed that they would have categorized the study as Not Human Subjects Research. Participants provided verbal informed consent, and participation was voluntary. No identifying information was collected. Those interested in accessing the dataset or replicating the analyses can contact the corresponding author.

### Study Population

The secondary data sets used in this study originated from a survey in Peking University Health Science Center. This survey was conducted in 217 dormitory rooms on campus. Investigators distributed questionnaires to each room and then collected them after completion. A total of 464 students aged 16–35 yrs from 185 rooms responded and 116 students did not finish survey questions. To assess peer effects at least two respondents per dorm room are needed. After excluding those only with one respondent per room, 419 students from 141 rooms were included in the final analysis.

In each room, one student was chosen at random to be the ‘index student’. We thus refer to the student randomly chosen as the ‘index student’ who is being influenced, and the others as the ‘peer(s)’. The original study was not powered for peer effects; however, for the purposes of examining peer effects we have an effective sample size of 141 rooms in our analysis. Our power to test each of the twenty one behaviors' association between index student and its peers ranged from 0.05 (frequency of vegetable intake) to 0.95 (Frequency of sweet food intake). Among those tests, eight of them (40%) have power >0.5, five of them (24%) have powers>0.8. Power and Sample Size Calculation version 3.0 [24] was used to calculate the power estimates above.

### Assessments and Measurements

The questionnaire consisted of four parts; Part I included questions on physical activity: location, frequency and time spent on moderate, vigorous and muscle-strengthening physical activity during the previous week, and use of a bicycle. Part II included questions on dietary habits, including intake frequency of vegetables (including whole juice), fruits (including whole juice), bean products (without soybean milk), coarse food grain (e.g. maize, millet), sweet food including candies/chocolate, sweet drinks, high-fat food, salty food and soybean milk (less than 1 day/wk, 1∼2 days/wk, 3∼4 days/wk, 5∼6 days/wk, almost every day); intake of fruits and vegetables (none, 1 serving/day, 2 servings/day, 3 servings/day, 4 servings/day and more than 5 servings/day); frequency of cooking method: deep fry, pan fry, steam/boil, fresh and roast/bake/toast (never, seldom, more or less, sometimes, always); and preference for eating in the school cafeteria or not. Part III included questions on smoking including smoking/second hand smoking status and number of cigarettes per day, etc. Part IV included questions on general information such as age, gender (male, female), school (Basic Medical Sciences, Clinical Medical Sciences, Nursing, Pharmaceutical Sciences, Public Health, Dental schools), ethnic group (Han, other), grade (undergraduate level from first year to fifth year; graduate level from first year to fifth year). At the end of survey, the investigator would record the corresponding building number (1–7), room number, number of roommates and assign a random ordered number to the respondents from the same room.

The survey questionnaire was designed to be answered within 25 to 40 minutes to ensure the quality of responses. The survey questionnaire was pilot-tested and revised to reduce unclear wording.

### Adjustment variables

Students were randomly assigned to roommates based on sex, school and school year. Analyses were thus adjusted for sex (male, female), school (Basic Medical Sciences, Clinical Medical Sciences, Nursing, Pharmaceutical Sciences, Public Health, Dental schools) and grade (Undergraduate 1–5 and graduate 1–5). Adjustment was also made for dormitory building 1–7 to help control for potential environmental confounding. Additional adjustment was also made after assigning indicators for the dormitory location (based on the surroundings gyms, play ground, food stores, etc): campus center or campus edge. Because roommates are assigned randomly to one another (i.e. randomly assigned to a particular cluster/dorm room) within strata of gender, major/school and year, any association between roommates' behaviors cannot be due to homophily/selection. Association between health behaviors of roommates must be due to either peer influence or environmental confounding. In this study we attempt to control for environmental confounding by adjusting for the building the students are in. In other studies of peer effects using roommate randomization [21], [22], pre-randomization characteristics are used to predict outcomes of roommates to circumvent environmental confounding but since such pre-college characteristics were not available in this study, environmental confounding was controlled for analytically.

### Data analysis

We conducted simple linear regression (treating categories as continuous variables) to examine the association between the index student's health behavior and corresponding peers' health behaviors using multivariate models adjusted for sex, school, grade, and dormitory location (campus center, campus edge) (Model 1). Restricted analysis was also conducted on those dormitories in the campus center due to the small number of rooms in buildings on the campus edge (Model 2). Since school is highly correlated with the dormitory building assignment, we also conducted analysis adjusted only for sex, dormitory building 1–7 and grade (Undergraduate 1–5 and graduate 1–5) (Model 3).

Note that provided that environmental confounding has been controlled for, under the null hypothesis of no peer effects, the correlation between roommates' behaviors should be zero. Moreover, missing data will not bias tests for peer effects because under the null hypothesis of no peer effects for any students in any dorm rooms, associations between outcomes between roommates should be zero in all rooms and for all students for which data are available. Missing data could bias estimates of peer effects but not tests.

We performed all analysis using SAS statistical software, version 9.2 (SAS Institute Inc, Cary, NC).

## Results

There were 419 students in the study population, with ages from 16 to 35. Among them, 59.2% were female students, 55.8% were undergraduate students, and most students were from the School of Basic Medical Sciences, School of Clinical Medical Sciences and School of Nursing (Data not shown); Among the 141 rooms, 58.9% were rooms with female students, and 97.2% of them are in the campus center (Table 1).

**Table 1:** Basic information for the study population.

Variable	N = 141	NO.	(%)
Sex	Male	58	41.1
	Female	83	58.9
Grade	Undergraduate 1	11	8.0
	Undergraduate 2	25	18.1
	Undergraduate 3	17	12.3
	Undergraduate 4	12	8.7
	Undergraduate 5	12	8.7
	Graduate 1	23	16.7
	Graduate 2	12	8.7
	Graduate 3	19	13.8
	Graduate 4	3	2.2
	Graduate 5	4	2.9
Building	Building 1	4	2.8
	Building 2	19	13.5
	Building 3	21	14.9
	Building 4	14	9.9
	Building 5	51	36.2
	Building 6	9	6.4
	Building 7	23	16.3
Building location	Campus edge	4	2.8
	Campus center	137	97.2
School	Basic Medical Sciences	41	29.1
	Clinical Medical Sciences	38	27.0
	Nursing	23	16.3
	Pharmaceutical Sciences	15	10.6
	Public Health	14	10.0
	Dental	5	3.6
	Others	5	3.6

For the prevalence of healthy behaviors, only 12.9% students were involved in vigorous-intensity exercise and 34.3% students involved in moderate-intensity exercise ≥3 days per week, while only 19.4% of them involved in muscle-strengthening activity. In addition, 49.9% students used a bicycle in daily life. For food intake, 70.9% of students ate vegetables almost every day, and 28.2% students had sweet foods ≥3 days per week. With regard to the preference of cooking methods, most student preferred pan-fried, steamed or boiled food. Around 94.4% students always ate in the school cafeteria; the smoking prevalence was 3.1% (Table 2).

**Table 2:** Heath behavior levels among the study population.

Health behavior	Level							
**Physical activity%**	None	1 day/week	2 days/week	3 days/week	4 days/week	5 days/week	6 days/week	7 days/week
Vigorous-intensity exercise (N = 417)	54.6	21.8	10.5	6.7	3.6	1.2	0.2	1.2
Moderate-intensity exercise (N = 417)	33.6	16.1	16.1	11.8	7.4	6.7	1.4	6.9
Muscle-strengthening exercise (N = 418)	80.6	8.1	3.4	2.9	2.9	0.0	0.2	1.9
**Vegetables and fruits intake amount%**	None	1 portion size	2 portion size	3 portion size	4 portion size	5 portion size		
Fruits (N = 417)	5.3	46.1	32.5	10.5	2.2	3.1		
Vegetable (N = 417)	2.4	25.8	44.9	15.5	7.9	3.1		
**Food intake frequency%**	<1 day/week	1–2 days/week	3–4 days/week	5–6 days/week	Almost everyday			
Vegetable including whole juice (N = 417)	3.1	7.2	9.3	9.1	70.9			
Fruit including whole juice (N = 411)	10.7	19.1	27.2	10.3	30.8			
Beans product excluding soybean milk (N = 415)	18.1	33	28.2	5.5	14.3			
Coarse food grain (e.g. maize, millet) (N = 416)	16.5	33.4	17.9	7.9	23.6			
Sweet food including candies, chocolate (N = 412)	33.7	36.6	16.5	5.7	6.0			
Sweet drinks (N = 415)	38.4	34.4	15.5	7.2	3.6			
High-fat food (N = 416)	31.5	38.0	20.3	5.5	4.1			
Salty food (N = 415)	43.9	31.7	13.6	5.0	4.8			
Soybean milk (N = 417)	31.5	32.2	17.2	8.4	10.3			
**Cooking method preference%**	Never	Seldom	More or less	Sometimes	Always			
Deep fry (N = 417)	4.8	44.6	34.6	15.0	0.5			
Pan fry (N = 411)	4.1	22.0	34.6	35.6	1.9			
Steam/boil (N = 414)	1.9	11.9	38.2	43.9	2.9			
Fresh (N = 412)	9.6	41.1	35.6	10.8	1.4			
Roast/bake/toast (N = 412)	10.5	37.5	35.8	13.8	0.7			
**Other behaviors%**	Yes	No						
Eat in the school cafeteria	94.4	5.6						
Smoking	3.1	96.9						
Use of bicycle	49.9	50.1						

For physical activity, peers' days of moderate-intensity exercise had a positive effect on the index student's corresponding behavior (Beta  = 0.24; 95% CI = −0.01, 0.49; p value  = 0.056); index student's bicycle usage was also significantly associated with his/her peers' bicycling usage (OR = 3.70; 95% CI = 1.36, 10.00, p value  = 0.010). For dietary intake, food preferences that were positively associated between the index student and that of the peers are sweet food included candies and chocolate, and roasted/baked/toasted food. Peers' eating in the school cafeteria was not statistically significantly associated with the index students' choices (OR = 0.87; 95% CI = 0.29, 2.60, p value  = 0.803) (Model 1, Table 3), but this may be due to the high prevalence of students eating in the cafeteria.

**Table 3:** Multivariate-adjusted point estimate and 95% confidence intervals for peer effects among students.

	Model 1		Model 2		Model 3	
Behavior	Diff (SE)	P value	Diff (SE)	P value	Diff (SE)	P value
**Continuous outcome**						
Vigorous-intensity exercise	0.10 (0.16)	0.528	0.07 (0.16)	0.668	0.15 (0.17)	0.369
Moderate-intensity exercise	0.24 (0.13)	0.056	0.22 (0.13)	0.079	0.22 (0.12)	0.078
Muscle-strengthening exercise	0.17 (0.16)	0.296	0.17 (0.17)	0.320	0.11 (0.16)	0.498
Food intake frequency						
Vegetable including whole juice	0.01 (0.11)	0.947	−0.03 (0.12)	0.835	−0.04 (0.12)	0.721
Fruit including whole juice	0.13 (0.10)	0.204	0.13 (0.11)	0.233	0.16 (0.10)	0.117
Beans product excluding soybean milk	−0.02 (0.10)	0.885	0.02 (0.11)	0.849	−0.002 (0.10)	0.988
Coarse food grain (e.g. maize, millet)	−0.08 (0.10)	0.470	−0.08 (0.11)	0.465	−0.07 (0.10)	0.524
Sweet food including candies, chocolate	0.36 (0.11)	0.002	0.39 (0.11)	0.001	0.33 (0.11)	0.004
Sweet drinks	0.19 (0.11)	0.097	0.20 (0.11)	0.084	0.13 (0.11)	0.244
High-fat food	0.14 (0.13)	0.300	0.12 (0.13)	0.381	0.09 (0.13)	0.481
Salty food	0.19 (0.12)	0.115	0.21 (0.12)	0.082	0.15 (0.13)	0.267
Soybean milk	0.19 (0.10)	0.065	0.16 (0.10)	0.128	0.19 (0.10)	0.049
Food intake amount						
Fruits	0.13 (0.11)	0.250	0.13 (0.11)	0.255	0.11 (0.11)	0.305
Vegetables	0.12 (0.12)	0.292	0.13 (0.12)	0.278	0.14 (0.12)	0.227
Cooking Method						
Deep fry	−0.003 (0.12)	0.979	−0.01 (0.12)	0.906	0.01 (0.12)	0.931
Pan fry	−0.02 (0.12)	0.879	−0.01 (0.12)	0.912	−0.03 (0.12)	0.798
Steam/boil	0.15 (0.11)	0.194	0.14 (0.12)	0.232	0.15 (0.11)	0.190
Fresh	0.08 (0.11)	0.497	0.10 (0.11)	0.366	0.06 (0.11)	0.557
Roast/bake/toast	0.39 (0.12)	0.001	0.37 (0.12)	0.002	0.37 (0.12)	<0.001
**Binary outcome (odds ratio)**	OR (95%CI)	P value	OR (95%CI)	P value	OR (95%CI)	P value
Use of bicycle	3.70 (1.36, 10.00)	0.010	3.66 (1.37, 9.80)	0.010	4.00 (1.55, 10.31)	0.004
Eat in school cafeteria	0.87 (0.29, 2.60)	0.803	0.69 (0.22, 2.15)	0.803	0.76 (0.25, 2.32)	0.627
Smoking	N/A	N/A	N/A	N/A	N/A	N/A

Model 1: Gender-, major-, grade-and building location-adjusted model;

Model 2: Gender-, major-, grade-adjusted model among those living in campus center;

Model 3: Gender-, grade-and building-adjusted model.

After restricting to those lived in the campus center, we obtained similar results as with model 1. For model 3 (when control was made for only for building, and not both school and building, which are correlated), soybean milk intake became significantly associated between index students and peers (Beta  = 0.19; 95% CI = −0.01, 0.39, p value  = 0.049) (Table 3).

Other behaviors including vigorous-intensity activity, vegetable & fruit intake did not show evidence of peer effects in our study (Table 3).

## Discussion

In our campus-wide study among Peking University Health Science Center students, the physical activity level was low among students; students had relatively high levels of vegetable intake and only few students smoked. We found evidence for health behavior peer effects at the dormitory room level, in particular, in the use of bicycles, moderate-intensity exercise, sweet food consumption, soybean milk intake (frequency), and roasted/baked/toasted food intake (cooking method).

Peer effects have been studied in multiple areas. Researchers have investigated peer effects of smoking, HIV related sexual behavior, alcohol use and drug use among adolescents in high school and among adults in community [16], [17], [18], [25], [26] as well as peer effects in academic achievement among students in elementary school or college [21], [22]. For example, Snow's study exploring the role of peer reputations and coping effects on cigarette smoking among 241 adolescent females attending the same school in Australia found evidence for the importance of coping with respect to cigarette smoking [27]. Sacerdote's study employed data from 1589 Dartmouth college students found that peers have an impact on grade point average and on decisions to join social groups, based on a similar randomized cluster-assignment design [23]. Based on their results, many researchers have recommended further peer-based interventions to improve health outcomes [20],[28].

A recent research study also examined the influence of peers on physical activity and dietary intake among children aged 9–11 years over a 2-year period. The study used a peer influence questionnaire and found peer influence on physical activity behavior but not dietary intake. For example: friends would talk with each other about how much they like physical activities, remind each other to be physically active, and also change their schedules in order to exercise together [29]. More recently, a well-designed follow-up study also found strong evidence of peer effects in weight gain among female students in college. The study showed that the amount of weight gained during the freshman year was strongly and negatively correlated to the roommate's initial weight, suggesting that female students adopt some of their roommates' weight-loss methods which could then cause them to gain less weight [23]. In conclusion, previous work showed evidence for the existence of peer effect on students' attitudes, and certain behaviors and health outcomes.

In our study, we also found peer effects on students' health behaviors. Our contribution to the literature is that peer effects on dietary habits and physical activity may exist among college/university students. College and university life is an important period for behavioral adaptation [23]. Compared to students from primary school or high school settings, roommates in college or university spend a lot of time together. They not only share the same learning environment, but also the same living space and social activity environment. Given the fact that students in the Health Science Center would attend many required classes and they only have a short period of winter (one month) and summer (two months) holidays, they are exposed to each others' behaviors almost every day, including eating and physical activity. In our study, students' moderate-intensity activity seems to be positively associated with their roommates' behavior. One potential explanation is that they may be involved in regular and easy-to-learn physical activities together, like running in the gym or on the playground.

As regards dietary intake, roommates likely share food within a room or go to school cafeteria together. Food recommendations from one individual could also have a potential effect on the other's choice. But based on our study, preference for eating in the school cafeteria didn't show significant peer influence but this may have been because the prevalence of eating in the school cafeteria was so high. Thus the positive peer effect on sweet food (including candies and chocolate) intake, and preference for roasted/baked/toasted food would be due to their food sharing in the room or their food shopping together, resulting in their similar food pattern preferences.

Another interesting finding in our study is the peer effect on bicycling among students. The odds of students' usage of bicycling increased 2.7 times if their roommate is bicycling. Bicycling, as an environmentally-friendly transportation approach, can increase the physical activity level which then could have significant benefits for health. There has been research showing that bicycling may be a better choice to increase physical activity compared to dancing and walking, since dancing involves discretionary time and many people walk too slowly to achieve efficient and effective exercise levels [30]. Our finding indicated that a peer-based strategy may be an effective intervention to improve bicycling.

There are several strengths in our study. To the best of our knowledge, this study is the first university level study to investigate health behavior peer effects within dormitory rooms. We evaluated a variety of health behaviors including physical activity, dietary intake and tobacco use. Physical activity was categorized into vigorous-intensity, moderate-intensity and muscle-strengthening activities according to the WHO recommendation criteria [31]. Bicycling was also included as one behavior since it is a popular commuting tool among university students in China. We also utilized a randomized cluster-assignment design to help eliminate the problem of peers selecting each other based on observable and unobservable characteristics, thereby providing more valid evidence of causality. Those individuals in our sample have randomly-assigned peers, and the adjustment of building locations helps control the potential confounding from the shared environment.

There are also limitations of this study. According to the random roommate assignment conditioning on sex and major, it is expected that their pre-randomization characteristics, for example, weights, are uncorrelated. However, the lack of data on characteristics prior to school entry makes us unable to check and confirm the randomization process. This is a limitation of our cross-sectional study design. In addition, the self-reported questionnaire approach also made our study prone to potential under/over estimation of individuals' health behaviors.

Overall, the utilization of the randomization design strongly supports the validity of our results. WHO has proposed to use low-cost methods to prevent chronic diseases, pointing out that schools and the community are important places for disease control and health promotion [32]. Based on our findings, we suggest that to improve health outcomes, physical activity and healthy dietary habits, promotion efforts need to incorporate peers in the process. Considering that students' unhealthy behavior is sometimes ignored by school, and also the difficulty of adopting healthy lifestyles, more attention should be given to students with unhealthy behaviors. Further research is necessary to understand which behaviors are most affected by peers and how to implement intervention strategies to address the growing burden of chronic disease. Most importantly, public actions with appropriate peer based approaches should be directed more intensively toward students with unhealthy lifestyles.
